# Detecting Morphing Attacks through Face Geometry Features

**DOI:** 10.3390/jimaging6110115

**Published:** 2020-10-29

**Authors:** Stephanie Autherith, Cecilia Pasquini

**Affiliations:** 1 Department of Computer Science, University of Innsbruck , Technikerstraße 21A, 6020 Innsbruck, Austria; stephanie.autherith@uibk.ac.at; 2 Department of Information Engineering and Computer Science, University of Trento, Via Sommarive 9, 38123 Trento, Italy

**Keywords:** face morphing, forensics detection, face landmarks, automatic border control

## Abstract

Face-morphing operations allow for the generation of digital faces that simultaneously carry the characteristics of two different subjects. It has been demonstrated that morphed faces strongly challenge face-verification systems, as they typically match two different identities. This poses serious security issues in machine-assisted border control applications and calls for techniques to automatically detect whether morphing operations have been previously applied on passport photos. While many proposed approaches analyze the suspect passport photo only, our work operates in a differential scenario, i.e., when the passport photo is analyzed in conjunction with the probe image of the subject acquired at border control to verify that they correspond to the same identity. To this purpose, in this study, we analyze the locations of biologically meaningful facial landmarks identified in the two images, with the goal of capturing inconsistencies in the facial geometry introduced by the morphing process. We report the results of extensive experiments performed on images of various sources and under different experimental settings showing that landmark locations detected through automated algorithms contain discriminative information for identifying pairs with morphed passport photos. Sensitivity of supervised classifiers to different compositions on the training and testing sets are also explored, together with the performance of different derived feature transformations.

## 1. Introduction

Automated face recognition and verification are widely studied problems in computer vision, for which accurate solutions have been developed and commercialized [1,2]. As a result, they are used in security contexts as means for person authentication, thus representing an alternative to more traditional schemes based on passwords and PINs (Personal Identification Number) and to other biometric traits like fingerprints. This includes applications such as face-based authentication in mobile devices and automated border controls (ABC) through passport photos [3].

In the ABC scenario, face information is used for identity verification starting from electronic Machine Readable Travel Documents (eMRTD). To this end, a live probe image of the subject physically present at border control is acquired and compared with the image stored in his/her eMRTD via face verification (FV) algorithms, which provide a binary output indicating whether the two images depict the same subject. In order to aid both algorithmic and human FV, photos in eMRTD must fulfil restrictive quality standards, as specified by the International Standard Organization (ISO) and the International Civil Aviation Organization (ICAO) guidelines. In particular, the face must be straight looking, acquired in frontal position, and not covered by hair or clothes.

**Figure 1:**
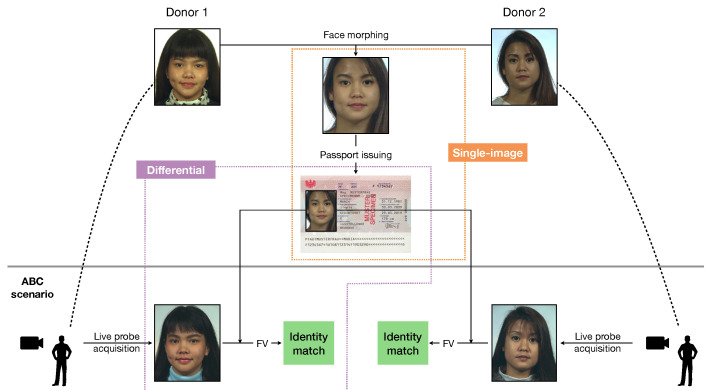
Illustration of a morphing attack against face verification (FV)-based automated border control (ABC) systems (examples are taken from the dataset in [5]): the area of analysis of single-image and differential approaches is highlighted.

Facilitated by these requirements, advanced FV algorithms can typically perform identity verification rapidly and accurately, but their effectiveness can be compromised if the images stored in eMRTDs contain alterations. A relevant case is represented by face images resulting from morphing operations [4], i.e., when two images of different subjects are blended together through geometric operations. In this case, FV algorithms are led to detect a match between the morphed image of the eMRTD and probe images from both subjects, as we illustrate in Figure 1.

In many countries, the image stored in the eMRTD is provided by the citizen during the passport application process, either in printed format or via web-platforms. This offers opportunities for an attacker to introduce altered visual information to be used to their advantage. In this context, a *morphing attack* would allow the same passport containing a morphed photo to be used by two different subjects, potentially including citizens with known criminal records for which border crossing would be forbidden. This kind of attack is particularly insidious as humans can be deceived as well with good probability, as it is shown in [6]. Moreover, it does not require physical forgeries of passports.

In order to contrast possible frauds exploiting these vulnerabilities, techniques for the detection of morphing attacks have been proposed in recent years.

The majority of them focuses on a *single-image* scenario, i.e., they analyse the photo in the eMRTD looking for traces of morphing operations. This includes inhomogeneities in texture patterns, camera fingerprints and compression traces, or visual artefacts like ghost shadows or illumination patterns. An advantage of this class of techniques is that they operate on eMRTD information only and could in principle reveal anomalies before the actual ABC context or even directly during the passport application process, thus enabling an early prevention of morphing attacks. However, they typically suffer from generalization issues due to the high variability of pre- and postprocessing operations which should be expected in real world scenarios [7]. In fact, as widely investigated in the field of image forensics, steps like compression [8], printing/scanning operations [9], resizing [10], and aspect ratio correction might be applied to the photo under investigation with highly diverse parameters and in turn introduce further subtle distortions and artifacts, which can have a strong impact on the (typically weak) morphing traces in the image signal [11,12].

Another interesting yet less explored approach is to consider a *differential* scenario, where the morphing detection is performed with the identity verification process at border control. In this case, the eMRTD photo and the live probe image can be jointly analyzed; thus, the decision is based on an *image pair*. While less timely than the single-image case in detecting anomalies, differential detection can leverage the additional information given by the acquired probe image.

In our work, we address this differential scenario and focus on the use of geometric face features to determine whether the image pair actually contains photos of the same subject or the reference eMRTD image depicts a morphed face. The rationale behind this choice is to capture the geometric inconsistencies between the morphed face and the genuine subject’s face that are unavoidably introduced in the morphing process. In fact, the morphing operation impacts the 2D face geometry, while its role has been only marginally investigated in the literature for morphing detection [13,14]. We fill this gap by developing and assessing the effectiveness of binary detectors based on the location of facial landmarks detected in both faces, the eMRTD photo, and the live probe. Those detectors are intended to be applied at ABC on top of the FV algorithm in cases where it detects an identity match between the two faces, since morphing attacks steer the FV decisions towards a positive match.

We can summarize our contributions as follows:

- We conduct an extensive experimental campaign to assess the effectiveness of landmark-based geometric features for the pairs. This includes adopting different training/testing conditions to encourage a sufficiently high variability between training and testing sets in terms of source datasets and subject characteristics and to better assess the generalization abilities of the detectors. A corpus of images belonging to different source datasets has been constructed, which represents a wider and more diverse benchmark with respect to previous studies in this direction [13,14].
- We identify the more relevant face areas for morphing detection through an ablation study on semantically related groups of landmarks, thus gaining insights on the face locations where more discriminative patterns can be found.
- We compare the performance of different transformations of the full set of facial landmarks, including feature representations previously proposed the literature [13,14] and geometric features stemming from findings in facial anthropometry.
- We evaluate the effect of noise sources that can typically affect the image pairs in realistic scenarios, revealing that the performance of the proposed detectors against unseen processing in the training tests are largely preserved. This confirms the advantage of geometric-based method of being stable against common image alterations, as opposed to texture-based approaches.

The manuscript is organized as follows: Section 2 reports an overview of existing approaches for face morphing creation and detection; in Section 3, we illustrate the detection framework and feature representations adopted; Section 4 fully reports the outcomes of the experimental tests we conducted; and Section 5 concludes the paper.

## 2. Related Work

We illustrate how morphed faces are created (Section 2.1) and then give an overview of the detection techniques proposed in the literature, differentiating between single-image (Section 2.2) and differential (Section 2.3) approaches.

### 2.1. Creation of Morphed Faces

**Figure 2:**
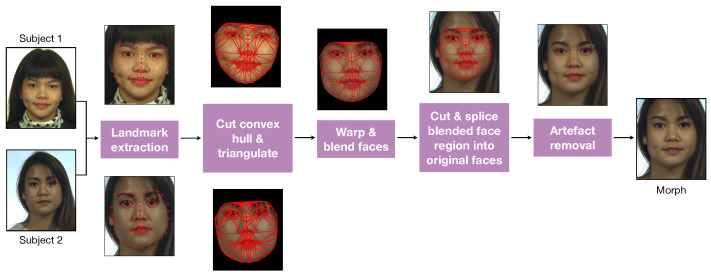
Visualization of the morphing process.

Face morphing consists of merging together two images depicting two different subjects (called *donors*) into one *morphed* face image, which contains characteristics of both subjects. This process generally involves several rule-based procedures and, although variants can be devised [15], we refer to the work in [6] and visually summarize the main steps in Figure 2.

Firstly, facial landmarks are detected in both images and linearly blended with a factor which is commonly set to 0.5 [6], so to obtain intermediate landmarks, which are subsequently triangulated. Then, both images are warped to be aligned to the intermediate landmarks and joined together again through cross-dissolving. This can be done on the entire image or by operating only on the convex hull of the landmark set to ease seamless alterations. Additional manual operations can then be applied to remove visual artifacts. Also, visually plausible morphs are generally possible provided that the subjects are depicted in a frontal pose and share similar characteristics, including the same gender.

While some tools are available online [16], obtaining high-quality full-face morphs that do not contain evident visual artefacts and that could then be used for potential attacks is highly time-consuming or requires specific software, generally proprietary [17] or not publicly available [6].

Given the impressive results obtained for other visual tasks, in [18], the authors attempt to use Generative Adversarial Models (GAN) to systematically create morphed faces, although generated images have a fairly low resolution. A follow-up study has been reported in [19], where a higher quality is reached, thus highlighting the potential advantages and promising outcomes of this approach.

### 2.2. Single-Image Detectors

The methods developed to detect morphing attacks on the reference eMRTD photo mostly rely on pattern recognition techniques used in image processing and image forensics. In fact, the key idea is to detect traces in the image signal of the operations involved in the morphing creation process.

Several approaches explored the effectiveness of texture and keypoint descriptors in detecting anomalies within the passport photo [20,21,22]. This includes Local Binary Patterns (LBP) [23], Binarized Statistical Image Features (BSIF), and Weighted Local Magnitude Patterns, also combined with other handcrafted features used in computer vision such as Scale Invariant Feature Transform (SIFT), Speeded Up Robust Features (SURF) [24], and Histogram of Oriented Gradients (HOG) [20].

Other methodologies resort to techniques originating from image forensics for the detection of local image modifications. To this purpose, a possible approach is to analyse the Photo Response Non-Uniformity (PRNU), which is an imperceptible spatial noise pattern caused by inaccuracies in the sensor manufacturing process. Every acquiring sensor has a characteristic PRNU, and alterations due to morphing can be revealed through its estimation [25,26]. Similarly, local modifications imply diversified compression histories within the same picture, which can be captured by analyzing proper statistical artifacts [15,27]. Also, traces of alterations can be found through modeling light reflection and light sources in different faces areas, observing whether they are physically consistent [28].

Recently, deep features have also been used for morphing detection, either by training or fine-tuning known architectures [29,30] or by using pretrained models as feature extractors . The advantage of neural networks is that they can in principle detect different kind of artifacts, although large datasets with high variance are necessary for training them successfully.

### 2.3. Differential Detectors

Differential detectors are less explored with respect to single-image methods, and few approaches appear so far in the literature.

One direction is explored by the work in [31,32], where the authors develop a pipeline to reverse the morphing process and to retrieve two face images starting from the one stored in the eMRTD. A morphing attack is detected if one of the two resulting face strongly matches the probe image.

Then, the works in [13,14] firstly combine information from facial landmarks detected in both images, and are further defined in Section 3.2, as they are considered as baselines in our tests. Therefore, the *directed distances* proposed in [13] constitute a transformation aimed at exposing shifting patterns in the landmark geometry. Those geometric artefacts are introduced by the warping step specifically in the morphing process. The features in [14] instead comprise *distances and angle differences* computed between landmarks of two face images. Herein, the angle differences are calculated between neighboring landmarks, while the distance features consider combinations of all the available landmarks. Finally, a solution building on deep face representations has been described in the recently published work [33].

## 3. Detection Framework

The analyzed geometry-based detectors operate in the presence of the eMRTD and the probe live image depicting the physical subject. As explained in Section 1, the detection is intended to be applied after the FV outcome if an identity match is detected.

**Figure 3:**
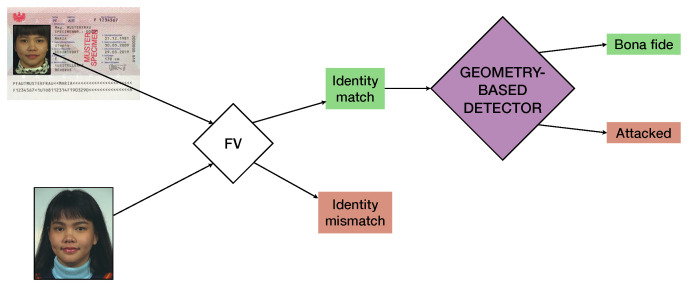
Detection framework.

In fact, advanced FV algorithms for ABC are designed and calibrated to robustly link faces belonging to the same subject, which are generally in frontal position with close-to-neutral expression but also contain common disturbance factors (such as differences in pose, illumination, and subject’s age/haircut). On the other hand, morphing attacks specifically challenge the FV’s ability to differentiate very similar yet strategically altered face geometries and thus to reject image pairs containing this kind of inconsistency. For this reason, the geometry-based detectors act as specialized modules based on facial geometry for the detection of potential morphing attacks among image pairs where an identity match results from the FV system, as depicted in Figure 3. Thus, the following classes of image pairs are used for training and testing:

- *Bona fide pairs:* the eMRTD contains a genuine face image of the physical subject.
- *Attacked pairs:* the eMRTD contains a morphed face image of which physical subject is a donor.

The geometry-based detector is a machine learning model that classifies the pair as either bona fide or attacked, based on the facial landmark information extracted from the two images. In Section 3.1, we describe the workflow adopted for the extraction and processing of the landmarks. Moreover, the extracted landmark vector L can be further combined and transformed through a function Φ to obtain derived feature representations Φ(L). This can be done in order to reduce the feature dimensionality (and thus to facilitate training also in the case of scarce training data) or to provide more interpretable outcomes, which is typically an advantage of handcrafted features. Thus, in addition to the full set of landmarks, we define different transformations of L inspired by studies in craniofacial anthropometry [34], the discipline that analyzes measurements and proportions of human faces.

### 3.1. Landmark Extraction

**Figure 4:**
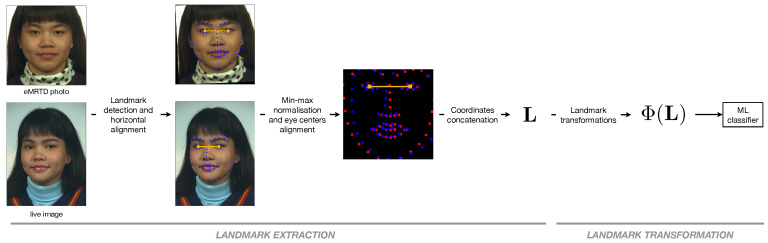
Landmark extraction and transformation.

Facial landmarks are biologically meaningful keypoints of human faces, widely used for many tasks in computer vision. Several algorithms have been proposed for the automatic detection and localization of these keypoints, and in our work, we use the dlib library, which outputs the coordinates of 68 landmarks as depicted in Figure 4. The eye centers are computed starting from the 6 landmarks of each eye, and the landmark coordinates are rotated so that the eye centers lie on the same horizontal line. After being mapped into the interval [0,1] through a min-max normalization, they are scaled in such a way that the two eye centers of each face are aligned.

The resulting vectors containing the bidimensional coordinates of the face in the passport photo and in the live image, respectively, are then concatenated together into a 68×2×2=272-dimensional vector L.

### 3.2. Landmark Transformations

In order to better encode in the feature vectors geometric characteristics of the two compared faces, handcrafted feature transformations can be applied to L. Here, we introduce for comparative testing (see Section 4) two different transformations inspired by anthropometric studies $\Phi_{R}$ and $\Phi_{A}$ (and their union), and we recall previously proposed landmark-based feature representations.

#### 3.2.1. Anthropometry-Based Features

**Figure 5:**
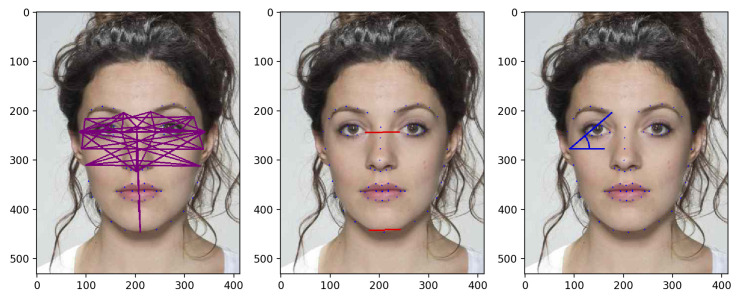
(**Left**) Forty-seven distances used in $\Phi_{R}$. (**Middle**) Two benchmark distances used in $\Phi_{R}$. (**Right**) Angle calculation as in $\Phi_{A}$.

Anthropometric craniofacial proportions [34] are characteristic ratios of distances between specific cranial and facial keypoints. They have been widely studied by anthropologists and used in different domains (ranging from art to medicine and from computer graphics to forensic sciences), and they have also been explored for 2D and 3D face recognition purposes [35,36]. We define the following transformations, yielding different features vectors:

- **Ratios ($\Phi_{R}$):** for each face, we consider 47 pairs of landmarks and compute the distance between them, as depicted in (Figure 5, left). Those landmarks are selected as highly involved in the morphing process and less sensitive to slight expression variations. Then, those distances are divided individually by the two benchmark distances depicted in red in (Figure 5, middle) and chosen so that they are reliably detected and relatively stable through the morphing process, according to the approach proposed in [36]. Those 94 ratio values from each face are then concatenated, resulting in a feature vector $\Phi_{R}\left(L\right)$ of size 188.
- **Angles ($\Phi_{A}$):** we take the 47 distances and the 2 benchmark distances used for $\Phi_{R}$ transformation. The angle between each of these distances and the horizontal line are then computed for the two faces (see Figure 5, right) and stored in a vector, resulting into a feature vector $\Phi_{A}\left(L\right)$ of size 49×2=98.
- **Ratios+Angles ($\Phi_{R}+\Phi_{A}$):** in this case, $\Phi_{R}\left(L\right)$ and $\Phi_{A}\left(L\right)$ are simply concatenated, the size of the feature vector being 188+98=286.

#### 3.2.2. Previously Proposed Landmark-Based Features

As mentioned in Section 2.3, previous approaches in morphing detection have utilized facial landmarks which consist of transformations of the vector L:

- **Directed Distances ($\Phi_{DD}$):** proposed in [13], the transformation yields a 136-dimensional vector containing shifting patterns between corresponding landmarks in the two faces.
- **All Distances and Neighbour Angles ($\Phi_{AD},\Phi_{NA}$):** the approach in [14] leads to two transformations: $\Phi_{AD}$ calculates a 2278-dimensional feature vector based on distances between all extracted landmarks of a face image; $\Phi_{NA}$ only considers angle differences between neighbouring landmarks and yields a 68-dimensional feature vector.

A common trait of these two landmark transformations is that they perform a one-to-one comparison of differente landmarks among the two faces, thus heavily relying on an accurate alignment of the two landmark sets. Instead, $\Phi_{A}$ and $\Phi_{R}$ process the landmark vectors separately for each face (ratios and angles are always computed within the same face) and then concatenate the two feature vectors of every pair. This mitigates potential inaccuracies of the alignment process, for instance, caused by slight pose variations.

## 4. Experimental Results

We now report the results of our experimental campaign, where the effectiveness of landmark-based geometric detectors is assessed. In Section 4.1, we describe the experimental setup adopted for our tests, including the datasets used, the machine learning classifier, and the evaluation metrics. Section 4.2 reports the results of our approach when the feature vector L containing all landmark locations is used for discrimination in different training and testing scenarios. An ablation study on different face areas is performed in Section 4.3, while in Section 4.4, we compare the different landmark transformation approaches described in Section 3.2. Finally, the robustness of the developed detectors in the presence of unknown processing in the testing phase is assessed in Section 4.5.

### 4.1. Experimental Setup

We used different datasets to create bona fide and attacked image pairs. Since most of the datasets were created for different tasks, in each case, we have selected images with frontal facing subjects exhibiting neutral expressions, according to the structure of each dataset. For the sake of clarity, in the following, we define multiple pair sets.

- Bona-fide pairs:
  - **AR**: 472 pairs formed starting from images in the AR dataset [37]. For every subject, pictures taken in two different acquisitions and distinct poses are available. We selected the 2 available frontal facing images where the face shows neutral expressions from both sessions and paired them with each other.
  - **REPLAY**: 140 pairs formed from frames extracted from the Replay dataset [38], which was originally proposed to benchmark detectors of face spoofing attacks.
  - **MISC**: a collection of 1000 pairs extracted from different datasets, including the Radboud Faces Dataset [39], the CVL Face Database [40], PUT Face Database [41], the FEI Face Database [42], and the Chicago Face Database [43].
- Attacked pairs:
  - **AMSL**: a total of 8700 pairs built from the publicly available AMSL Face Morph Image Dataset [44] used in [11]. A subset **AMSL**${}_{1000}$ is also determined by randomly selecting 1000 pairs from **AMSL**.
  - **FERET**: 4306 pairs composed from a dataset of morphed images released by Biometix [5]. The morphs have been created starting from images of the Feret database [45], which includes multiple acquisitions of the same subject.

Those sets will be differently combined for creating the training set TR (i.e., the union of bona fide and attacked training pair sets TR${}_{\mathrm{BF}}$ and TR${}_{A}$)and the testing set TS (i.e., the union of bona fide and attacked testing pair sets TS${}_{\mathrm{BF}}$ and TS${}_{A}$) for supervised machine learning models, as described in the following subsections. The operator |·| will indicate the number of pairs contained in each set.

In each test, an SVM classifier with radial basis function (RBF) kernel has been used for classification. The parameters gamma and *C* of the SVM have been selected via grid-search over a logarithmic grid ranging from $10^{-4}$ to $10^{1}$ for each dataset composition. Note that we have focused on the RBF kernel as it always outperformed linear and polynomial kernels in our tests. All the experiments have been performed in Python 3 and the scikit-learn, OpenCV, and dlib packages.

Consistently with other works in this domain, we adopt the metrics defined for the detection of presentation attacks in biometrics to measure the performance of the classification (i.e., thresholding the SVM score at 0):

- *APCER* (Attack Presentation Classification Error Rate): ratio of attacked pairs erroneously classified as bona fide pairs;
- *BPCER* (Bona fide Presentation Classification Error Rate): ratio of bona fide pairs erroneously classified as attacked pairs;
- *ACC* (Accuracy): fraction of image pairs that are correctly classified (either as bona-fide or attacked)

In addition, for selected cases, we show the Detection Error Tradeoff (DET) curve plotting *APCER* vs. *BPCER* obtained by varying the decision threshold on the output score of the SVM, and we will report the *EER* (Equal Error Rate), i.e., the error rate at the operating point where *APCER = BPCER*.

### 4.2. Full Landmark Set

We first test the effectiveness of the feature representation given by the full set of facial landmarks extracted from both images, i.e., the vector L.

We consider different experimental scenarios, always arranging the pairs in such a way that no subject appearing in the testing set is part of any pair used in the training set, not even as a donor of one morphed face. This is in fact the case for real-world applications where we cannot expect the identities in the testing phase to be present in the training set. To this purpose, we define a splitting procedure to form training and testing groups, where we select a part of the subjects appearing in a certain pair set and isolate all the pairs that contain those subjects. Note that the attack pairs consist of a morph and a probe image of one of its donors which was preferably not used during the morphing process. Thus, each morph yields at least 2 attack pairs with 2 images of its different donors. Given p∈[0,1] and a set SET, the following steps are performed:

1. a fraction *p* of the subjects appearing in SET are randomly chosen;
2. all the pairs in SET which depict any of these subjects in one or both images or as donors of a morphed fac, are stored in SET(p)
3. the remaining pairs in SET are stored in $\bar{\mathrm{SET}(p)}$

This procedure has been used to create TR and TS by varying *p*. In particular, we consider three scenarios differing for the composition of TS, as described in Table 1. The bona fide pairs are the same for each row, and the share of **AR** between training and testing varies with *p*. In the first two scenarios, the attacked pairs in TR${}_{A}$ and TS${}_{A}$ are drawn from the same pair set. In the third more challenging scenario, TS${}_{A}$ is composed by 1000 **AMSL** pairs plus a number of **FERET** pairs (depending on *p*), while only **FERET** pairs are tested; thus, only a fraction of training samples are from the same set as the testing samples. By doing so, we can observe how performance are affected by the numerosity and composition of TR and TS.

**Figure 6:**
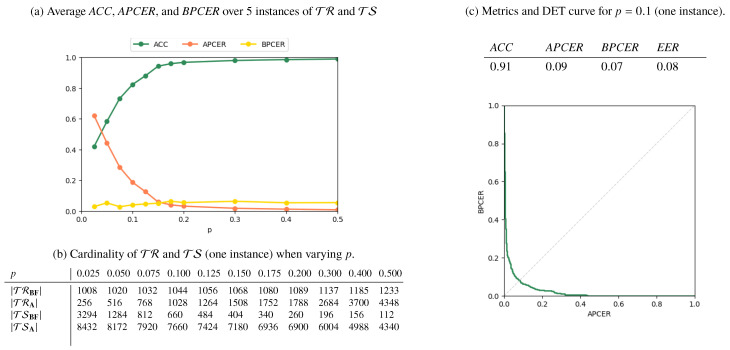
Results for the **AMSL**-*only* scenario.

**Figure 7:**
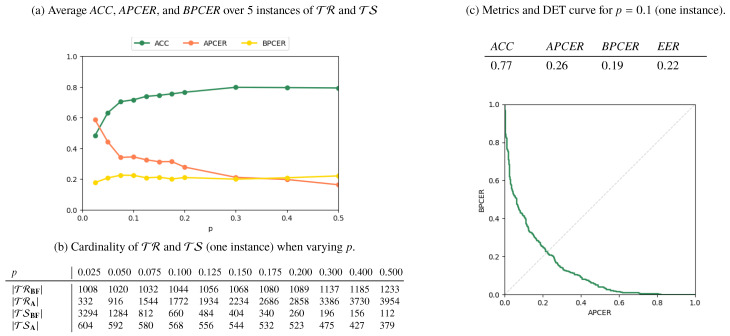
Results for the **FERET**-*only* scenario.

**Figure 8:**
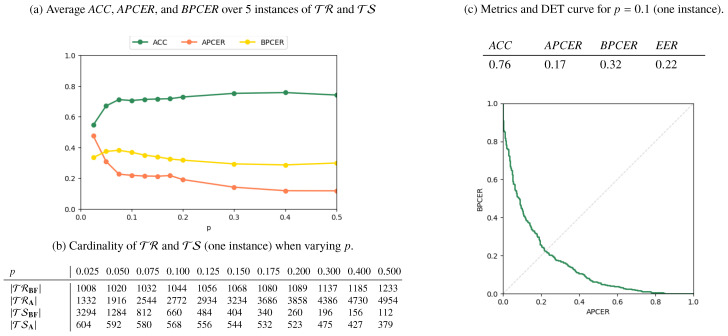
Results for the *Mixed* scenario.

Results for the **AMSL**-*only*, **FERET**-*only*, and *Mixed* scenarios are reported in Figure 6, Figure 7 and Figure 8, respectively. In Figure 6a, Figure 7a and Figure 8a, we plot the metrics *ACC*, *APCER*, and *BPCER* for different values of *p*. Since step a of the splitting procedure involves a random choice of a fraction *p* of subjects (for which the images are then included in TR), the metrics are averaged over 5 different splitting instances for validation. Figure 6b, Figure 7b and Figure 8b report the cardinality of the resulting training and testing groups for each class on a single splitting instance, for which we report in Figure 6c, Figure 7c, Figure 8c the DET curve and the performance metrics at p=0.1.

As expected, the performance increases with *p*, i.e., when more numerous and representative training samples are available. Overall, the best results are obtained in the **AMSL**-*only* scenario, with a global accuracy approaching 1 for p>0.2. The **FERET**-*only* scenario instead shows a lower accuracy, which stabilizes at around 0.77 even when *p* increases. This is explained by the higher variability of acquisition conditions of the probe images in the **FERET** pairs, which makes it harder to discriminate face geometry anomalies due to variabilities in the probe images or due to morphing operations, thus causing increased *APCER*, *BPCER*, and *EER*.

Finally, the *Mixed* scenario results show that, when trained mostly on the 1000 **AMSL** pairs, the detector struggles in recognizing **FERET** attacked pairs and performs well when samples in TR${}_{A}$ are roughly equally splitted in **AMSL** and **FERET** pairs. This suggests that different datasets carry peculiar characteristics and, as it typically happens for supervised machine learning solutions, there exists a risk of overfitting on specific sources.

For the sake of completeness, we have also investigated the use of a 1D convolutional neural network (CNN) classifier to better process the information contained in the landmark vector. We considered an architecture with 4 1-dimensional convolutional layers with a kernel size of 3 and [64,128,256,256] filters each. Succeeding the second convolution, we apply instance normalisation after every feature extraction layer. Before the classification, we apply a dense layer with 128 neurones.

In Table 2, we provide a comparison between the RBF SVM and the 1D CNN classifier for the *Mixed* scenario and p=0.1 (one instance) both in terms of performance and training/testing time. In fact, we report the average training time over different values of *p* and the average prediction time for the two classifiers; for the SVM, tests have been conducted on a 2.3 GHz 8-core Intel Core i9, while the CNN was trained and tested on an NVIDIA GTX 1080Ti GPU.

As it can be observed, the gain in performance with respect to the RBF SVM is rather marginal, in front of a much higher computational effort. We therefore employ the RBF SVM for the following analyses. Moreover, for the sake of brevity, we will stick to the *Mixed* scenario and the case p=0.1.

### 4.3. Ablation Study

**Figure 9:**
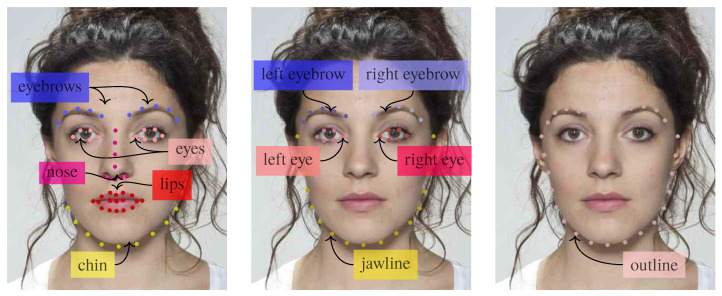
Landmark groups for our ablation study.

In order to determine the importance of different landmarks, we group them into distinct semantic groups, as shown in Figure 9, and observe their detection results. These groups correspond to facial attributes and are inspired by the semantic landmark groupings in [46].

We separately test landmarks corresponding to different semantic groups for each image pair and concatenate them to obtain a feature vector ${\mathrm{sL}}_{g}$, where *g* indicates a single semantic group or a combination of them. We then feed ${\mathrm{sL}}_{g}$ to a RBF SVM, just like we did for L.

The results are reported in Table 3. It can be observed that L is generally better performing. However, ${\mathrm{sL}}_{outline}$ achieves comparable results, thus suggesting that most of the relevant geometric information resides in the relative position of the face line and the eyes.

Moreover, it is worth noticing that the accuracy drop of different variants of ${\mathrm{sL}}_{g}$ is mostly due an increase in *BPCER* while the *APCER* remains rather low. This bias towards false alarms might be due to the selected features being less distinctive and the training set not containing enough information for characterizing non-attacked samples, so that bona fide pairs likely exhibit unseen patterns at testing time and are classified as attacked.

### 4.4. Comparison of Landmark Transformations

We now compare the performance of the different feature representations derived from the landmark location vector L introduced in Section 3.2, comparing them with other transformations proposed in the literature.

**Figure 10:**
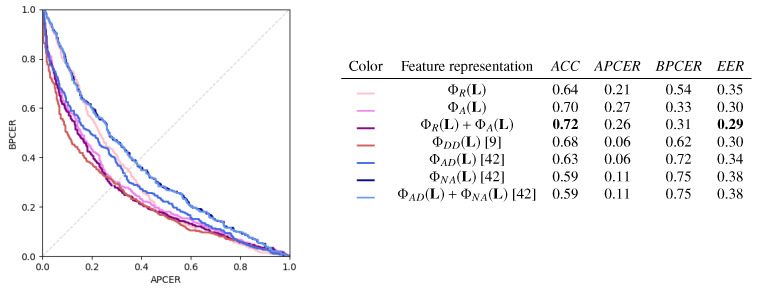
Results for different feature representations.

We report the results for the case of p=0.1 in Figure 10. On the left, the DET curves for the different transformations are reported, and for each of them, the performance metrics are reported on the right. Higher *ACC* and *EER* are obtained by the anthropometry-based transformations $\Phi_{R}+\Phi_{A}$ and $\Phi_{A}$. In general, the angle-based features in $\Phi_{A}\left(L\right)$ are more informative than the ratio-based ones in $\Phi_{R}\left(L\right)$, although their dimensionality is lower than all the other considered transformations. $\Phi_{DD}$ also has competitive performance, but its *APCER* and *BPCER* are strongly unbalanced. $\Phi_{AD}$, $\Phi_{NA}$, and their combinations yield less accurate classifications.

However, note that all feature representations underperform with respect to L. This suggests that, in the considered experimental scenario, the SVM model is powerful enough to learn effective classification boundaries directly in the feature space of L, and further, handcrafted transformations are not beneficial in terms of global accuracy.

### 4.5. Robustness to Processing Operations

We assess the robustness of our detector in the case of diverse processing operations applied to the eMRTD photo. This is in fact known to be a typical issue of for previous detectors, especially single-image ones, as passport pictures can undergo several operations in its digital history (e.g., printing/scanning and compression). To this purpose, we run our models also on different variants of the testing set, where selected postprocessing operations are applied to the passport photos as listed and described in Table 4.

**Figure 11:**
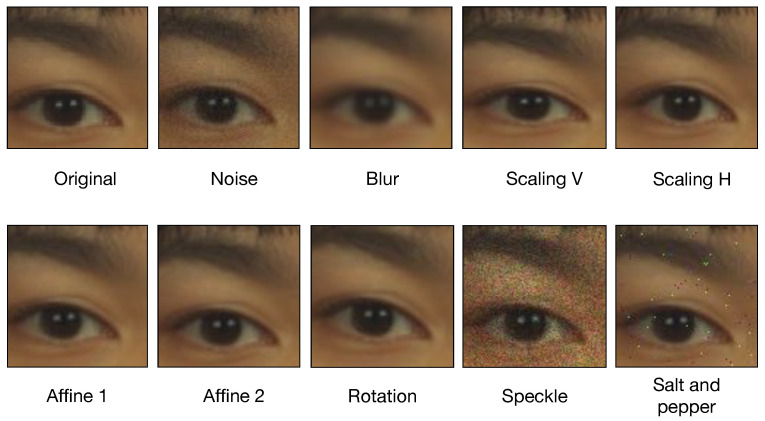
Example of processed electronic Machine Readable Travel Documents (eMRTD) pictures with different manipulations.

Examples of the different processing operations are reported in Figure 11, where a portion of the image is magnified.

In each case, we measure the performance loss with respect to the baseline case, where neither training nor testing underwent any processing. If *ACC* is the accuracy in the baseline case and *ACC${}_{P}$* is the accuracy when a certain processing P is applied to the testing set, we calculate the accuracy loss as

(1) $${ACC}_{\mathrm{Loss}}=ACC-{ACC}_{P}.$$

**Figure 12:**
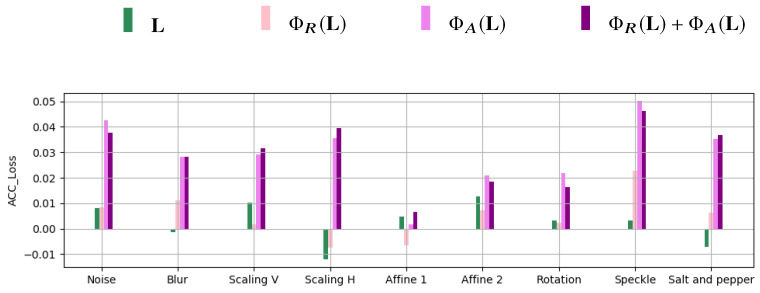
Values of *ACC*${}_{P}$ for different processing operations and feature representation.

Figure 12 reports ${ACC}_{\mathrm{Loss}}$ for each processing operation and for the feature representations L, $\Phi_{R}\left(L\right)$, $\Phi_{A}\left(L\right)$, and $\Phi_{R}\left(L\right)+\Phi_{A}\left(L\right)$.

We can see that the accuracy loss is always below 5% and involves mostly angle-based feature representations. The loss for the full landmark feature vector L is however very small (always below 2%) and essentially oscillates around 0. We can then conclude that the trained models generally preserve their effectiveness also in the presence of these unseen processing operations appearing in the testing set.

## 5. Conclusions

We have addressed the problem of detecting morphed faces in electronic passports at border control in a differential scenario, i.e., by jointly analyzing the photo contained in the electronic passport and the live probe image acquired on site. In doing so, we have performed a comparative analysis of geometric face features by developing detectors based on the facial landmarks and by exploring their effectiveness in different directions.

In different scenarios, best results are obtained by operating directly in the feature space of the 2D coordinates of the 68 facial landmarks extracted from the two face images of the pair under investigation. The performance remains essentially stable even when the testing samples are modified via processing operations that are unseen in the training phase. This confirms the advantage of relying on geometric cues like landmarks, for which extraction is generally reliable even after visual alterations that are not too impactful.

Moreover, ablation tests suggests that a non-processed full set of landmark coordinates provides more discriminative information in every case. Among the compared handcrafted features, the ones based on facial anthropometry concepts are generally more effective with respect to approaches previously proposed in the literature.

The obtained results confirm the potential of a geometric differential analysis leveraging also the probe image for detecting morphing attacks. The extracted features are indeed limited in dimensionality (thus are lighter to process with respect to more computationally expensive techniques [32]), while offering fair detection performance and high interpretability of the detector’s outcome. This is an advantage with respect to other differential detection approaches based on deep networks [33], which do not explicitly look for geometric distortions that are inherent to morphing attacks but rather rely on the distribution of deep features used for general face-recognition problems. However, our study also exposes typical issues affecting supervised machine learning techniques, namely the risk of overfitting training data and reduced generalization abilities when different data sources are tested. Multi-clue detectors would in fact be recommended for improved performance in realistic scenarios. In fact, a promising direction for future work would be to analyze geometric cues in conjuction with richer representations like the ones based on deep networks [33], which has brought a significant performance boost in many related tasks.

## Figures and Tables

**Table 1:** Training/testing scenarios adopted in Section 4.2.

	TR ${}_{\mathrm{BF}}$	TR ${}_{A}$	TS ${}_{\mathrm{BF}}$	TS ${}_{A}$
**AMSL**-*only*		AMSL(p)		$\bar{\mathrm{AMSL}(p)}$
**FERET**-*only*	MISC∪AR(p)	FERET(p)	$\bar{\mathrm{AR}(p)}\cup \mathrm{REPLAY}$	$\bar{\mathrm{FERET}(p)}$
*Mixed*		${\mathrm{AMSL}}_{1000}\cup \mathrm{FERET}\left(p\right)$		$\bar{\mathrm{FERET}(p)}$

**Table 2:** Performance metrics of different classifiers: the average training time is computed as the mean of training times over distinct values of *p*. We define the average prediction time as the mean of the time (measured over 100 examples) that it takes for our models to classify one image pair.

Model	*ACC*	*APCER*	*BPCER*	Average Training Time per *p*	Average Prediction Time per Pair
RBF SVM	0.76	0.17	0.32	0.69 min	0.0031 s
1D CNN	0.77	0.19	0.29	36.08 min	0.1768 s

**Table 3:** Results for the ablation tests.

Feature Representation	*ACC*	*APCER*	*BPCER*	*EER*
L	0.67	0.17	0.32	0.22
${\mathrm{sL}}_{\mathrm{eyes}}$	0.37	0.07	0.77	0.39
${\mathrm{sL}}_{\mathrm{left}\;\mathrm{eye}}$	0.11	0.06	0.93	0.40
${\mathrm{sL}}_{\mathrm{right}\;\mathrm{eye}}$	0.25	0.05	0.85	0.42
${\mathrm{sL}}_{\mathrm{eyebrows}}$	0.51	0.07	0.73	0.38
${\mathrm{sL}}_{\mathrm{left}\;\mathrm{eyebrow}}$	0.32	0.01	0.89	0.35
${\mathrm{sL}}_{\mathrm{right}\;\mathrm{eyebrow}}$	0.36	0.02	0.85	0.41
${\mathrm{sL}}_{\mathrm{eyebrows}\;+\;\mathrm{eyes}}$	0.52	0.16	0.57	0.34
${\mathrm{sL}}_{\mathrm{left}\;\mathrm{eyebrow}\;+\;\mathrm{eye}}$	0.37	0.08	0.77	0.36
${\mathrm{sL}}_{\mathrm{right}\;\mathrm{eyebrow}\;+\;\mathrm{eye}}$	0.46	0.09	0.75	0.39
${\mathrm{sL}}_{\mathrm{nose}}$	0.21	0.11	0.83	0.43
${\mathrm{sL}}_{\mathrm{lips}}$	0.44	0.15	0.48	0.30
${\mathrm{sL}}_{\mathrm{chin}}$	0.20	0.05	0.79	0.39
${\mathrm{sL}}_{\mathrm{jawline}}$	0.41	0.06	0.69	0.34
${\mathrm{sL}}_{\mathrm{outline}}$	0.64	0.09	0.38	0.23

**Table 4:** Manipulations applied for the robustness test.

Name	Description
Noise	Additive Gaussian noise with σ=0.5
Blur	Blurring with normalized box filter
Scaling V	Downscaling the vertical dimension by 1–2%
Scaling H	Downscaling the horizontal dimension by 1–2%
Affine 1	Applying small offsets to three selected landmarks and the corresponding affine transform to the whole image
Affine 2	Applying a small offset to one selected landmark and the corresponding affine transform to the whole image
Rotation	Rotating the image by ±3% degrees
Speckle	Multiplicative noise
Salt and pepper	Punctual noise on 4% of pixels
